# Comparative Reaction Modelling and k-Nearest Neighbors Analysis of *Cocos nucifera* Shell Thermal Degradation

**DOI:** 10.3390/polym18091070

**Published:** 2026-04-28

**Authors:** Abdulrazak Jinadu Otaru, Zaid Abdulhamid Alhulaybi Albin Zaid, Abdulrahman Salah Almithn, Ige Bori, Obinna Onyebuchi Barah

**Affiliations:** 1 Department of Chemical Engineering, College of Engineering, King Faisal University, Al Ahsa 31982, Saudi Arabia; zalhulaybi@kfu.edu.sa (Z.A.A.A.Z.); aalmithn@kfu.edu.sa (A.S.A.); 2 Department of Mechanical Engineering, Kabale University, Plot 364, Kabale Municipality, Kabale P.O. Box 317, Uganda; 3 Department of Mechanical Engineering, School of Engineering and Applied Sciences, Kampala International University, Kampala P.O. Box 20000, Uganda

**Keywords:** coconut shell, pyrolysis kinetics, third-order model (F3), machine learning, kNN

## Abstract

This study presents a definitive framework for *Cocos nucifera* (coconut) shell valorization, integrating high-resolution thermogravimetry with advanced machine learning. Physicochemical analysis confirms a high-energy feedstock (45.7% carbon, 71.5% volatiles), with SEM/XEDS and FTIR revealing heterogeneous, lignocellulosic, catalytic-rich structural matrix. TG/DTG analysis identified distinct degradation windows: hemicellulose (135–395 °C), cellulose (270–430 °C), and protracted lignin decomposition (275–675 °C). Kinetic modeling indicates that pyrolysis follows a third-order (F3) continuous degradation mechanism across the studied range, supported by high correlation coefficients ($R^{2}$ = 0.93–0.96). The mean kinetic and thermodynamic parameters—specifically an activation energy of 165 kJ·mol^−1^ (calculated across the 10–60 wt% conversion range during hemicellulose and cellulose pyrolysis), a positive activation enthalpy (159 kJ·mol^−1^), and a Gibbs free energy of activation (155 kJ·mol^−1^)—suggest that the thermochemical conversion of coconut shell is an endothermic, non-spontaneous process with moderate energy requirements. Furthermore, the integration of *k*NN machine learning yielded near-perfect predictive metrics ($R^{2}\approx 1.000$) using optimized hyperparameters (k=85 for TG, k=100 for DTG, and k=50 for conversion). These findings suggest that coconut shells can be efficiently valorized as a high-energy feedstock, with data enabling reliable and optimized prediction of thermal degradation to minimize experimental waste.

## 1. Introduction

The United Nations Sustainable Development Goal 7 (SDG 7) and the Saudi Green Initiative (SGI) establish frameworks aimed at achieving affordable and clean energy by 2030. These frameworks emphasize the diversification of the energy mix, the reduction in over-reliance on fossil fuels, and the promotion of efficient waste management and environmental sustainability. The Kingdom of Saudi Arabia’s (KSA) policy regarding the exploration of bioenergy derived from biomass, particularly agricultural waste, is informed by Vision 2030. This policy promotes significant research initiatives focused on converting biomass waste into usable biofuels, while also targeting environmental sustainability through effective solid waste management and the reduction in greenhouse gas emissions [1]. Consequently, understanding the bioenergy potential of biomass waste—whether through experimental research or predictive modeling—is vital for the realization of sustainable energy strategies, the mitigation of climate change, and the implementation of effective waste management practices (the 4R’s: reduce, reuse, recycle, and recovery). This approach aligns with the principles of a circular economy and resource efficiency.

The bioenergy potential of various agricultural wastes has been extensively documented in the literature, with most of these studies focusing on the quantification of biomass mass loss during thermal processing. This approach facilitates the analysis of key compositional components and the application of kinetic models to estimate potential reaction mechanisms, as well as to predict the quality and quantity of products generated from biomass conversion processes. For example, El-Sayed et al. [2] conducted a thermokinetic analysis of palm fronds (PFs), olive leaves (OLs), and wheat straw (WS) utilizing thermogravimetric analysis (TG) data collected at three distinct heating rates—10, 20, and 30 °C·min^−1^—and a degradation temperature range extending from ambient conditions to 1000 °C. The estimated activation energies for the thermal degradation of hemicellulose, cellulose, and lignin determined over a conversion range of 0.2 to 0.8 using the Flynn–Wall–Ozawa (FWO) and Kissinger–Akahira–Sunose (KAS) regular integral isoconversional methods were found to be 91.9, 69.1, and 65.2 kJ·mol^−1^ (FWO) and 87.5, 101.8, and 63.4 kJ·mol^−1^ (KAS), respectively. The calculated Gibbs free energy of the activated complex (∆G) reported in the study indicated that greater thermal energy is required to depolymerize the biomass materials into simpler monomers and oligomers.

Uddin Monir et al. [3] assessed the bioenergy potential of coconut shell using TG data collected over a temperature range of 30 to 1000 °C at a heating rate of 5 °C·min^−1^ under an inert N_2_ atmosphere. Thermokinetic evaluation of TG data via the Coats–Redfern method indicates an activation energy ($E_{A}$) of 68.9 kJ·mol^−1^ and a pre-exponential factor (A) of 0.1 min^−1^ for the primary pyrolysis stage within the 315–600 °C range [3]. These findings characterize the kinetic parameters of the active devolatilization phase during the pyrolysis of the coconut shell. Values reported for the activation enthalpy (∆H), Gibbs free energy of the activation (∆G), and the entropy of activation (∆S) are 65.2 kJ·mol^−1^, 193.1 kJ·mol^−1^, and −0.3 kJ·mol^−1^·K^−1^, respectively. Although this study [3] provides valuable insights into the pyrolysis of coconut shell, the application of the Coats–Redfern model-fitting kinetic method at a single heating rate has been discouraged by the International Confederation on Thermal Analysis and Calorimetry (ICTAC) [4], which recommends the use of model-free kinetic methods across multiple heating rates as more efficient and reliable for estimating kinetic data.

Several studies related to the bioenergy potential of agricultural wastes have been documented in the literature, including those focusing on walnut shells [5], oil palm waste [6], woody biomass [7], and mustard stalks [8], among others. Research in the field of thermochemical conversion of biomass materials has also expanded to include modeling and simulation, particularly through the application of machine learning algorithms. For instance, [9] employed four distinct machine learning algorithms—namely artificial neural networks (ANNs), decision trees (DTs), k-nearest neighbors (kNN), and random forest (RF)—to predict the mass loss profiles derived from the thermal devolatilization of oxidatively torrefied spent coffee grounds (SCGs). The RF model was reported to exhibit the highest accuracy ($R^{2}\sim 0.998$) for both the training and testing (unseen) datasets. Similarly, Faroque et al. [10] implemented sixteen distinct regression models to predict the thermogravimetric analysis (TGA) traces obtained from the thermal decomposition of biocomposites, which were produced by incorporating chemically treated (alkali NaOH treatment) wheat straw residual fillers into an epoxy resin matrix. Their findings indicated that kNN regression outperformed the other fifteen (15) regression models, achieving a coefficient of determination value of 0.999. Furthermore, an ANN-based machine learning model was utilized to predict the influence of hemicellulose, cellulose, and lignin on the pyrolysis kinetics of 32 types of biomasses at varying conversion rates, ranging from 0.1 to 0.9 [11]. The optimal number of hidden neurons was reported to be between 7 and 11 [11], with deviations between the modeled and experimental data for hemicellulose, cellulose, and lignin degradation being 14.1%, 20.8%, and 12.9%, respectively.

While numerous machine learning models have been utilized to predict and optimize biomass pyrolysis kinetics and TG traces, research specifically focusing on the direct application of the k-nearest neighbors (kNN) algorithm for analyzing thermogravimetric data from coconut shell thermal devolatilization is limited. Building on the proven success of machine learning models in analyzing other biomass types, this study explores the potential of kNN for coconut shell pyrolysis. Despite the extensive literature on biomass pyrolysis kinetics, only a limited number of studies have been conducted in strict adherence to the International Confederation for Thermal Analysis and Calorimetry (ICTAC) recommendations for reliable thermogravimetric (TG) data acquisition and kinetic analysis. Stringent experimental protocols are essential for accurate kinetic parameter estimation, which include adhering to a maximum heating rate of 20 °C·min^−1^, maintaining sample weight variations within 10% between experiments, and ensuring the product of sample weight and heating rate remains below 100 mg·°C·min^−1^ to mitigate heat and mass transfer limitations [4,12]. Furthermore, achieving high-reliability in kinetic estimation necessitates conducting multiple TG runs at different heating rates (minimum of three), allowing for the application of model-free isoconversional methods. This study provides a comprehensive assessment of the thermal devolatilization behavior of *Cocos nucifera* (coconut) shells, featuring the novel application of the k-nearest neighbors (kNN) machine learning model to predict and optimize TG, derivative thermogravimetry (DTG), and conversion profiles. The investigation was conducted through the following structured objectives:

1. Feedstock Characterization: Comprehensive assessment of coconut shell through proximate and ultimate analysis, coupled with morphological and chemical analysis utilizing SEM/XEDS and FTIR.
2. Thermal Analysis: Investigation of the non-isothermal pyrolysis of coconut shell at multiple heating rates, in strict compliance with ICTAC recommendations to ensure data reliability.
3. Kinetic Mechanism Analysis: Deconvolution of DTG traces using Bi-Gaussian functions to resolve overlapping peaks and estimate the lignocellulosic composition (hemicellulose, cellulose, lignin) and constituent-specific fuel yields.
4. Thermokinetic Investigation: Elucidation of biomass pyrolysis kinetics, thermodynamic parameters, and reaction mechanisms via model-fitting and model-free isoconversional methods applied to non-isothermal thermogravimetric data.
5. Predictive Modeling: Application of kNN algorithms to pyrolytic kinetic data for the prediction and optimization of TG, DTG, and conversion profiles.

Integrating thermokinetic and kNN machine learning models is crucial for maximizing the bioenergy potential of coconut shell pyrolysis. The key difference lies in approach: reaction modeling offers mechanistic insight into the underlying kinetics (“why”), while kNN uses historical data to predict specific outcomes (“what”). This combined strategy is expected to provide insights into the production and optimization of valuable bioenergy carriers (biofuel, biochar, biogas) and to streamline the design and scale-up of industrial reactors. Secondly, this methodology will support comparative analyses across various lignocellulosic biomass feedstocks to determine optimal conversion routes and specific high-value uses, such as biocatalyst acceleration in biopolymer systems or soil fertility enhancement. Collectively, this is expected to advance sustainable waste management practices and promote the transition to a circular economy.

While the existing literature confirms the successful application of various machine learning and deep learning models to the analysis of biomass waste pyrolysis, the kNN model was specifically selected as the preferred choice for this study. This preference is attributed to several key advantages: its simplicity (an intuitive and straightforward algorithm to implement), ease of hyperparameter tuning, low computational demands, high interpretability, and robust performance with small- to moderately sized datasets [13]. This reliable modeling approach facilitates the optimization of operating conditions for maximizing bioenergy potential, circumventing the need for costly and time-consuming physical experiments.

## 2. Research Methodology

**Figure 1:**
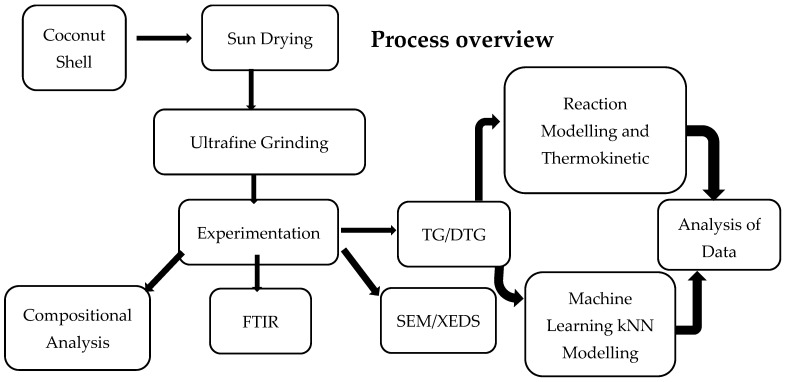
Experimental and modeling process overview.

Figure 1 illustrates an overview of the experimental and modeling process, delineating the sequential utilization of coconut shell as a feedstock for pyrolysis operations. This process commences with material pre-treatment and pulverization, followed by the experimental characterization of the fine powdered sample. Subsequently, the analysis of experimental data is conducted through reaction modeling, thermokinetic analysis, and machine learning k-nearest neighbors (*k*NN) modeling.

### 2.1. Pre-Treatment of Biomass Material

The primary material examined in this study was *Cocos nucifera* shell (CCS), which was sourced from Al Ahsa, located in the Eastern Region of Saudi Arabia. The CCS material was sun-dried for a period of 20 days to reduce its moisture content, increase its energy density, facilitate the breakdown of complex structures, and enhance the yield of valuable products during pyrolysis operations [14]. The dried sample was subsequently milled into a powder and sieved to obtain a desired particle size range of 75–300 µm for characterization. The pulverization process involved a two-stage size reduction: initial crushing of the shells into smaller chunks, followed by ultrafine grinding (pulverizing) using a BN801ME Ninja Blender (1200 W). The final product was then sieved to achieve the required fineness. The selection of this narrow size range is based on minimizing the effects imposed by thermal contact surfaces [15], as smaller particles exhibit less internal resistance to heat and volatile gas release [16], resulting in a more rapid devolatilization of biomass material during pyrolysis operations.

Both the apparent and compressed densities of the sample were measured at 0.5 g·cm^−3^ and 0.6 g·cm^−3^, respectively, which fall within the lower expected range of apparent densities (0.5–0.6 g·cm^−3^) for milled coconut shell [17], and can be attributed to the extended drying time of the biomass sample. Following the density measurements, several additional experimental analyses, including proximate and ultimate analyses, Fourier transform infrared spectroscopy (FTIR), scanning electron microscopy (SEM), energy-dispersive X-ray spectroscopy (EDS), and thermogravimetric analysis (TGA), were conducted on the milled dried sample of the CCS biomass, and the data obtained from these analyses were subsequently evaluated.

### 2.2. Proximate and Ultimate Analysis

The proximate analysis of the CCS sample, determining its moisture, volatile matter, ash, and fixed carbon contents, was conducted using a TG analyser in accordance with [5,18]. The TG analyser measures weight changes over specific temperatures and time intervals to estimate these components. A 10 mg sample of CCS biomass underwent thermo-oxidative heating from room temperature at a constant rate of 10 °C⋅min^−1^ to enable distinct decomposition stages and accurate analysis [19]. Moisture, volatile matter, and carbon residue contents were determined via sequential isothermal holds: 105 °C for 1.5 h (moisture), 600 °C for 8 min (volatile matter), and 900 °C for 1.5 h (ash). Fixed carbon percentage was calculated by subtracting the sum of these components from 100%, as defined by the formula: Fixed Carbon%=100%−(Moisture%+Volatile Matter%+Ash%).

The ultimate analysis of the CCS biomass was performed using a VELP Scientifica EMA 502 Elemental Micro Analyzer with dual carrier gases (Helium/Argon), following a procedure like that in [20]. A 2.3 mg sample, wrapped in an aluminum crucible, was combusted in an oxygen-rich atmosphere within the analyser. The elements were converted into their respective gases (CO_2_, H_2_O, N_2_, SO_2_). Helium carrier gas transported the products through a gas chromatography (GC) column for separation. A thermal conductivity detector (TCD) measured the quantity of each gas produced, and the data was subsequently processed and analyzed.

### 2.3. SEM/XEDS Procedure

The scanning electron microscopy (SEM) and energy-dispersive X-ray spectroscopy (EDS) measurements of the milled CCS biomass were conducted by capturing images of the material surfaces to analyze its morphology, microstructure, and elemental composition using the Joel JCM-700 NeoScope SEM system. A similar experimental procedure to that described in [21] was employed during this measurement. Prior to this analysis, the CCS material was mounted on a stub and coated with gold–palladium to enhance the emission of secondary electrons, thereby improving the signal-to-noise ratio and facilitating the enhancement of image clarity [22]. The prepared sample was subsequently placed onto the sample stage within the sample chamber of the SEM system, followed by the application of the JCM-7000’s advanced automatic functions to obtain high-quality images of the surfaces of the CCS biomass. The chemical elements present on the surface of the CCS biomass materials were also detected and recorded with the assistance of the EDS detector, which is fully integrated into the SEM system.

### 2.4. FTIR Procedure

The identification of functional groups and molecular compositions present in the CCS biomass sample was conducted using an Agilent Cary 630 Fourier transform infrared (FTIR) spectrometer, equipped with Agilent MicroLab software (version 5.8). This analysis involved measuring light absorption by the material across a wavenumber range of 500 to 4500 cm^−1^ [23,24]. A resolution of 4 cm^−1^ was used, with 74 scans taking a total measurement time of approximately 30 s. This resolution provides a reasonable balance between obtaining detailed information to distinguish key spectral features and maintaining a short measurement time.

The methodology employed for FTIR measurement commenced with the cleaning of the crystal surface (sample position) of the spectrometer using acetone to facilitate the acquisition of reliable data, thereby preventing interference from contaminants and ensuring an accurate spectral background. The auto sample presser of the FTIR analyser was securely positioned against the clean crystal surface to measure the background of the attenuated total reflectance, with the objective of isolating and removing any interfering signals, thereby yielding a final spectrum that is representative solely of the sample. A small quantity of the milled CCS biomass sample was subsequently applied to cover the crystal section of the spectrometer and was firmly held in place by the presser. Upon initiation, the sample absorbed beams of infrared light at various frequencies, resulting in the unique spectrum that was recorded by the software.

Furthermore, the resulting FTIR data were used to estimate the lateral order index (LOI), hydrogen bond index (HBI), and total crystallinity index (TCI), the latter of which is also referred to as the total order index (TOI). These indices quantify the structural arrangement and crystallinity of cellulose—the primary component influencing bio-oil yield—and its reactivity toward thermal degradation [25]. The LOI of the CCS biomass was determined using the absorbance ratio of the bands at 1429 cm^−1^ and 898 cm^−1^. These peaks correspond to CH_2_ scissoring (indicative of crystalline regions) and C-O-C stretching at the β-glycosidic linkage (representative of the amorphous phase), respectively [26]. The HBI was estimated as the ratio of the broad O–H stretching absorbance (typically 3300–3400 cm^−1^) to a reference peak, such as the CH_2_ bending (e.g., around 1320 cm^−1^ for CH_2_ bending) [25,27]. Finally, the TCI was calculated as the ratio of the crystallinity-sensitive peak at 1370 cm^−1^ (peak sensitive to crystallinity) to the C-H stretching peak (2884 cm^−1^ in this study), representing total material volume [27].

### 2.5. TG/DTG Procedure

Thermogravimetric data for the pyrolysis of milled CCS biomass were acquired using a Mettler Toledo TGA/SDTA 851e TGA analyzer. The experimental procedure followed that reported in [21], with strict adherence to the ICTAC recommendations for kinetic studies [12]. The TG measurements were conducted at heating rates of 5, 10, and 20 °C·min^−1^, with degradation temperatures ranging from 25 to 1000 °C. A 4.0 ± 0.1 sample of the powdered CCS biomass was accurately placed in the TG analyser’s crucible, ensuring that the sample uniformly covered the bottom of the crucible. This was done to facilitate representative reactions across the sample, prevent thermal lag, and improve the reliability and reproducibility of the pyrolysis data [28]. The selected sample mass was maintained within ±10% of the mean weight, ensuring gravimetric uniformity and adherence to the standard heuristic rule reported in [12], which requires the product of sample weight and heating rate to remain below 100 mg·°C·min^−1^. The crucible containing the biomass material was then positioned within the heating chamber of the TG system, which was rendered inert by the introduction of nitrogen gas (N_2_) at a flow rate of 20 mL.min^−1^. The temperature range and heating rate of 5 °C·min^−1^ were entered into the data acquisition software (STARe Software Version 15.00, Build 8668), followed by the measurement of the sample weight within the software.

Upon initiation of the experiment, sample weight loss as a function of degradation temperature was measured and recorded in the software. The operation continued until the maximum input temperature of 1000 °C was reached. The heating chamber of the TG system was then allowed to cool before conducting subsequent experiments to prevent thermal damage to the equipment and to ensure accurate and reproducible data, thereby mitigating the impact of residual heat on subsequent measurements [29]. The experiment was conducted in triplicate using similar TG procedure. The mean weight losses and associated standard deviations for triplicate experimental runs at heating rates of 5, 10, and 20 °C min^−1^ were 34.2 ± 0.9 wt.%, 38.5 ± 0.3 wt.%, and 45.5 ± 0.8 wt.%, respectively. The low data dispersion indicates high precision and experimental reproducibility. Furthermore, the observed trend of increasing weight loss with higher heating rates aligns with established biomass thermal degradation behavior [30].

The DTG data for the thermochemical conversion of the CCS biomass material were calculated by taking the ratio of differences between sample weight loss and degradation temperature obtained from the TG profiles. Deconvolution of the DTG traces was performed using a custom MATLAB script (Version 2025a) to isolate pseudo-components corresponding to the degradation pathways of moisture, hemicellulose, cellulose, and lignin. The MATLAB deconvolution code employs a bimodal Gaussian mixture (Bi-Gaussian) kernel to account for the potentially asymmetric shape of the peaks. This kernel is defined by four parameters: two means (*μ*_1_, *μ*_2_) and two standard deviations (*σ*_1_, *σ*_2_). Initial values for peak amplitude, peak width, and half-width were specified based on the actual DTG peaks. An interactive optimization algorithm, specifically the Levenberg–Marquardt least squares curve-fitting function, was utilized to fit the model parameters to the convolved data [31]. A trial-and-error approach involving initial guesses of the peak parameters (amplitude, width, and half-width) for each lignocellulosic component was implemented until the individual deconvoluted signals closely matched the raw DTG data. The resulting deconvoluted data were subsequently exported to Microsoft Excel for further analysis. The deconvoluted profiles were analyzed to determine the lignocellulosic fractional composition of the CCS biomass using Simpson’s 1/3 rule (Equations (S1) and (S2), Supplementary Data). Furthermore, the yields of the thermochemical conversion products—bio-oil, biochar, and syngas—were calculated using the stoichiometric and empirical relations defined in Equations (S3)–(S7) (Supplementary Data).

### 2.6. Reaction Mechanisms and Thermokinetic Equations

The estimation of kinetic triplet and thermodynamic parameters for the thermal decomposition of CCS biomass material commences with a thorough understanding of the governing equations pertinent to their estimation. These thermokinetic equations are presented in the Supplementary Data of this study. The kinetic triplet comprises the potential reaction mechanism(s) for the formation of activation complexes, the activation energy ($E_{A}$) and the pre-exponential factor (A). The thermodynamic parameters include the activation enthalpy (∆H), the Gibbs free energy of the activation complex (∆G), the entropy of activation (∆S) and the equilibrium constant (k). The Coats–Redfern (CR) model-fitting method (Equation (S8)) [32] was employed to evaluate the pyrolysis kinetics of CCS biomass across heating rates of 5, 10, and 20 °C·min^−1^. To identify the most probable reaction mechanisms, 26 distinct solid-state kinetic models were tested, as detailed in Table S1 [21]. These models are categorized by their underlying physical geometry and rate-controlling steps, including geometrical contraction (GCM), reaction-order (ROM), power law (PLM), diffusion (DFM), sigmoidal rate (SRE), and Carter models. Also, the integrating reaction models with combined CR methods facilitated the determination of kinetic compensation parameters via Equation (S10), which, in turn, enabled the estimation of the pre-exponential (A) factor for activated complex formation using Equation (S11) [33,34].

The activation energy (E) for the formation of activated complexes was determined using several isoconversional methods, including the Standard Flynn–Wall–Ozawa (FWO) (Equation (S12)) [21,35,36] and its fourth-order Senum–Yang Iterative variant (Equations (S13) and (S14)) [37]. Additionally, the Kissinger–Akahira–Sunose (KAS) (Equation (S15)), Vyazovkin method (Equation (S16)) [38], and the Friedman (Equation (S18)) [5,39,40] method were employed. These models facilitate the estimation of robust kinetic parameters without requiring a predefined reaction mechanism [40]. Conversely, the Friedman kinetic model is classified as a model-free differential method, facilitating a direct assessment of how the activation energy varies with conversion [40], without necessitating prior knowledge of the specific solid-state reaction model. It is essential to reiterate that the selection of model-free kinetic methods for the estimation of kinetic parameters in this study is based on recommendations outlined in the ICTAC committee report published in [4], which provides detailed information regarding the analysis of kinetic data. The report [4] emphasizes the reliability of model-free kinetic methods in comparison to model-fitting kinetic methods and specifies the requirement of a minimum of three heating rates (TG traces) for the estimation of kinetic parameters, contrasting with single heating rate model-fitting kinetic methods. Equations (S19)–(S22) present mathematical models for the estimation of activation enthalpy (∆H) [20], Gibbs free energy of activation complex (∆G) [38,41], entropy of activation (∆S) [41,42], and equilibrium constants (k) [42].

### 2.7. Machine Learning kNN Approach

The application of the k-nearest neighbors (kNN) algorithm in this study was employed for the prediction and optimization of the thermogravimetric analysis (TGA) traces obtained from the pyrolysis of CCS biomass. The selection of this supervised learning algorithm is attributed to its capacity to manage non-linear and complex relationships within the data without imposing strong assumptions [43]. This characteristic renders it particularly suitable for modeling the intricate chemical processes encountered in the thermochemical conversion of biomass, exemplified by the TG traces analyzed in this study. Machine learning kNN is categorized as a lazy learner [43] or distance-based algorithm that does not necessitate a training phase; instead, it identifies “k” nearest training examples and classifies them based on averaging (for regression, as in this study) or majority voting (for classification) [44,45]. The accuracy of the model is contingent upon the estimated k-value utilized, which functions as a hyperparameter throughout the learning process. In summary, a low k-value may result in overfitting, while a high k-value may lead to underfitting, thereby producing oversimplified decision boundaries, elevated bias, and inaccuracies.

The prediction of TG, DTG, and conversion profiles for the pyrolysis of milled CCS biomass was implemented using a kNN algorithm, developed in MATLAB™. Initial data pre-processing involved categorizing the datasets into features (inputs) and an output (label). Specifically, the input parameters consisted of heating rate, time, and temperature, while the outputs comprised percentage weight loss derived from TG, DTG, and conversion data. These operating parameters are selected for their direct influence on the kinetic, mass transfer, and thermodynamic mechanisms of coconut shell decomposition. Heating rate determines the energy flux, governing internal thermal lag [46] and providing essential data for high-speed reactor modeling. Pyrolysis temperature serves as the thermodynamic threshold for bond dissociation, regulating degradation stages and bioproduct distribution [47]. Finally, residence time—which is intrinsically linked to temperature—facilitates secondary cracking of volatiles, shifting the product yield from bio-oil toward non-condensable gases [48]. To maximize predictive accuracy, each output parameter was trained independently using a dedicated kNN model, as a single, unified model would likely fail to capture the distinct, specialized relationships within the TG/DTG, conversion data. By isolating individual kNN models, each instance can independently optimize its hyperparameters—specifically the neighborhood size (k) and distance metric—to align with the unique statistical characteristics of its respective data stream [49,50]. The selected input parameters are considered the primary operational conditions that influence decomposition process [51]. Consequently, they directly affect the resulting weight loss profile, which is why they were chosen as input parameters for the kNN modeling in this study.

Each input variable or dataset consisted of 588 data points, which were selected based on a systematic sampling of data selection using a temperature interval of 5 °C. All experimental data were assessed to ensure the absence of outliers and categorical data. A 70/15/15 data splitting strategy was employed, utilizing 70% (412 data points) of the data for training, 15% (88 data points) for validation, and 15% (88 data points) for testing. This type of data splitting strategy is justified because it provides a large enough training set (70%) to model complex biomass kinetics, a dedicated validation set (15%) for precise hyperparameter optimization of TG/DTG peaks, and an independent test set (15%) to ensure the model generalizes accurately to new experimental data without overfitting [52]. While TG and conversion profiles exhibit inherent structural continuity, the DTG traces provide more granular insights into biomass degradation stages, including moisture removal and the decomposition of hemicellulose, cellulose, lignin, and char. Despite the unstructured nature of DTG data, the density of the 588-point dataset allows a kNN model to precisely map these distinct thermal regions. By utilizing 412 training points (via a 70/15/15 split), the model effectively captures the fundamental pyrolysis kinetics of coconut shell, ensuring high predictive accuracy on unseen test data. Min-Max normalization was applied to the three data subsets to scale features into a specific range, typically [0, 1]. This pre-processing technique is essential for preventing features with larger magnitudes from dominating the model, thereby facilitating improved training performance and accelerated convergence [53].

To optimize the kNN model, a hyperparameter grid search was conducted to determine the ideal configuration for the number of neighbors (k), the distance metric, and the weighting scheme. The search space was defined as the interval [1, 100]. Evaluated distance metrics included Euclidean, Manhattan (City Block), Chebyshev, Minkowski, and Hamming distances. Additionally, three weighting schemes were assessed: Uniform (Equal), Inverse Distance, and Squared Inverse Distance. This methodology utilizes a systematic and exhaustive search to mitigate the inherent weaknesses of distance-based models—specifically sensitivity to noise, attribute scaling, and neighborhood size—thereby accommodating the distinct kinetic characteristics of coconut shell pyrolysis and enhancing the accuracy of weight loss and conversion rate predictions [50,54]. The kNN function in MATLAB was subsequently applied to the training datasets to generate a predictive model capable of replicating both the training, validation and testing datasets. To demonstrate model reliability, statistical metrics were computed between predicted and experimental data. Furthermore, Pearson correlation matrices were analyzed to determine feature sensitivity regarding TG, DTG, and conversion outputs.

## 3. Results and Discussion

### 3.1. Proximate and Ultimate Analysis Data

Table 1 presents the proximate analysis of the powdered CCS material, detailing its percentage compositions of moisture, fixed carbon, volatile matter, and ash, which are 6.3%, 17.8%, 72.3%, and 3.6%, respectively. Although the estimated moisture content falls slightly below the 7.4–11.0% range recommended by Irawan et al. [55], this lower value is advantageous, as it reduces the energy demand for pre-pyrolysis drying. Furthermore, lower feedstock moisture generally enhances the calorific value of the resulting bio-oil by minimizing water dilution in the liquid product [56]. The high volatile matter content of 72.3% suggests that the CCS biomass material is energetically efficient for ignition and has potential for releasing combustible gases and vapor during thermochemical conversion [57]. The estimated fixed carbon content for the CCS biomass (17.8%) is within the moderate limit of the range (10–30%) [58] specified for biomass pyrolysis. This moderate fixed carbon content indicates a high proportion of volatile matter, which could be harnessed for energy. The estimated ash content of 3.6% falls the 2–5% range reported for woody biomass [59] and is well within the broader 0.1–67% range for biomass [60]. Consequently, this material can be classified as having a medium ash content, making it a viable feedstock for thermochemical conversion.

Table 1 presents the ultimate analysis data for the CCS biomass material, indicating carbon (C), hydrogen (H), nitrogen (N), sulfur (S), and oxygen (O_2_) compositions of 45.7%, 4.9%, 2.8%, 0%, and 46.6%, respectively. The combination of higher percentages of carbon and oxygen in the dried sample suggests that the CCS material is characterized by substantial proportions of oxygen-rich hemicellulose and cellulose, which decompose at lower temperatures, and carbon-rich lignin [61], which decomposes at higher temperatures, thereby contributing significantly to the solid biochar produced after the thermochemical conversion process. The hydrogen composition of 4.9% in the CCS biomass feedstock is notably low, indicating a limited availability of hydrogen atoms to facilitate the formation of hydrogen-rich gases during pyrolysis [62]. This situation may potentially result in increased CO and CO_2_ formation from the thermal devolatilization of carbonyl and carbonyl groups present in the lignocellulosic biomass material. The low nitrogen content of 2.8% characterizing this material falls within the moderate range (0.3–6.0%) [63] of acceptable values, suggesting lower emissions of nitrogen oxides (NOx) during the pyrolysis operation of the CCS biomass material. Furthermore, Table 1 indicates that there are no traces of sulfur present in the CCS biomass, suggesting that such material will not emit sulfur-based pollutants during its thermochemical conversion, which is remarkably advantageous in achieving cleaner and more sustainable forms of energy.

### 3.2. Analysis of SEM/XEDS Data

**Figure 2:**
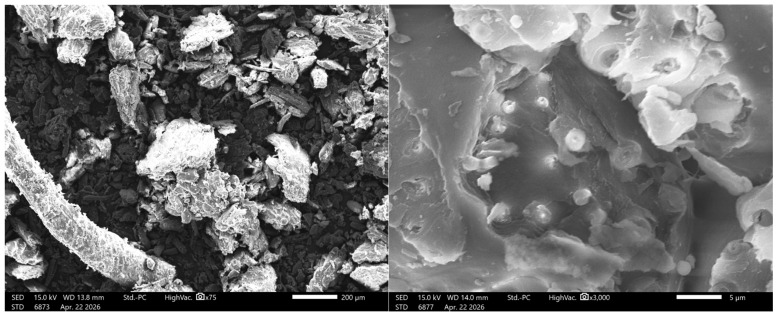
SEM micrographs of the milled CCS biomass at ×75 (**left**) and ×3000 (**right**) magnifications.

As shown in Figure 2, SEM images of dried and pulverized coconut shell reveal a heterogeneous, lignocellulosic structure that strongly influences its pyrolysis behavior. At low magnification (×75, left), the material shows irregular, angular particles with fibrous fragments and dense agglomerates, reflecting fracture along cellulose microfibrils embedded in a rigid lignin matrix. This confirms the highly lignified and compact nature of coconut shell biomass [64]. At higher magnification (×3000, right), rough surfaces, microcracks, pits, and globular features are observed. These correspond to collapsed cell lumens, intercellular voids, and lignin-rich or extractive domains. Lignin’s amorphous structure promotes the formation of such rounded features, while microcracks enhance internal accessibility [65].

These structural characteristics imply limited initial porosity and diffusion constraints, leading to slower heat and mass transfer during pyrolysis. However, the high lignin content enhances thermal stability and promotes higher char yields, as lignin decomposes over a wide temperature range [66]. The observed microcracks and pits act as precursors for pore development during devolatilization, making coconut shell an excellent precursor for biochar and activated carbon [67].

The SEM-observed dense, lignin-rich morphology of coconut shell is consistent with the XEDS composition (C = 49.34%, O = 39.05%) in Table 2, which reflects a typical lignocellulosic framework dominated by carbonized biopolymers. The relatively high oxygen content yields an O/C mass ratio ≈ 0.79, indicating abundant oxygenated functional groups (e.g., hydroxyl, carbonyl) associated with cellulose and hemicellulose. This supports the SEM evidence of limited porosity and compact structure, as oxygen-rich polymers promote strong intermolecular bonding and structural rigidity [66].

According to Mohan et al. [65], during pyrolysis, a high O/C ratio favors devolatilization and bio-oil formation, producing oxygenated compounds such as phenols, acids, and aldehydes, but reduces energy density of the resulting fuel. As temperature increases, progressive deoxygenation lowers the O/C ratio, enhancing aromaticity and stability of the resulting biochar [65]. The presence of inorganic elements (K = 4.27%, Ca = 2.53%, Na = 2.64%, P = 1.50%) indicates significant ash-forming minerals. Alkali and alkaline earth metals (AAEMs), particularly K and Ca, act as catalysts for pyrolysis, promoting char formation, cracking reactions, and gas evolution [64]. These minerals also facilitate pore development, complementing the microcracks observed in SEM. However, they may reduce bio-oil yield and influence slagging behavior at high temperatures. While present in trace quantities, magnesium (Mg = 0.4%) serves as a moderate catalyst that facilitates char formation and the stabilization of carbonaceous structures during coconut shell pyrolysis, albeit with lower catalytic efficacy than K or calcium (Ca). In contrast, chlorine (Cl) primarily undergoes volatilization as hydrogen chloride (HCl); while this contributes to equipment corrosion and alters ash chemistry, its direct catalytic influence on the process is negligible [64,68].

Overall, while structural heterogeneity may cause non-uniform heating, the CCS biomass remains a highly suitable pyrolysis feedstock due to its high carbon content, stability, and pore-forming potential. Combined SEM-XEDS analyses demonstrate that the biomass is mineral-rich and moderately oxygenated, enhancing char yield, catalytic reactivity, and pore development. Consequently, CCS biomass is an ideal precursor for producing high-quality biochar and activated carbon.

### 3.3. Analysis FTIR Data

**Figure 3:**
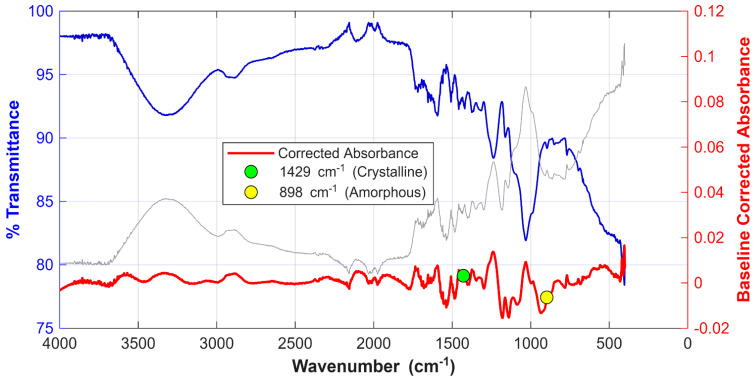
FTIR spectrum of the CCS biomass showing percentage transmittance [%] and baseline corrected absorbance against wavenumber [cm^−1^].

An FTIR spectrum of the CCS biomass is illustrated in Figure 3, plotting percentage transmittance and absorbance against wavenumber [cm^−1^]. The broad peak at 3309 cm^−1^ is indicative of O-H stretching associated with hydroxyl groups, likely originating from cellulose, lignin, and absorbed water [24,69]. In contrast, the peak at 2884 cm^−1^ falls within the absorption band of 2800–2900 cm^−1^, corresponding to the stretching vibrations of CH, CH_2_, and CH_3_ groups [70] present in the lignocellulosic biomass. The peak observed at 2096 cm^−1^ falls outside the standard spectral range for typical functional groups found in raw lignocellulosic biomass; however, it might point to an unusual C=O (carbonyl overtone) or could be an artifact related to sample preparation or the presence of a specific inorganic impurity [71]. The prominent peak at 1593 cm^−1^ is highly suggestive of aromatic C=C bonds within the material’s aromatic rings, a characteristic feature frequently associated with the presence of lignin [72,73]. In addition, the peak at 1370 cm^−1^ falls within the 1300–1400 cm^−1^ region, which is often assigned to a mix of aliphatic CH, CH_2_, and CH_3_ bending modes related to lignin or cellulose structures [70].

The peak at 1719 cm^−1^ signifies the presence of acetyl groups, commonly found in hemicellulose, lignin, pectin, and other oxygen-containing functional groups [73]. The peaks at 1236 cm^−1^ and 1030 cm^−1^ fall within the fingerprint region. While this region is generally less suitable for precise molecular identification [74,75], these specific peaks are nonetheless indicatives of several key structural components. The peak 1236 cm^−1^ may indicate the presence of C-O stretching in ester groups and aromatic ethers, which are predominantly found within the complex structure of lignin, while the peak 1030 cm^−1^ may suggest the presence of C-O-C glycosidic linkages of oligosaccharides or C-O deformation in primary alcohols [76].

The estimated LOI, calculated as the absorbance ratio of the peaks at 1429 cm^−1^ and 898 cm^−1^ [26], was found to be 0.507. This estimated LOI value indicates that the coconut shell has a moderate-to-low level of crystalline order compared to chemically treated coconut shell fibers, which can reach values as high as 0.8 or more [73,77]. This suggests that the coconut shell used in this study contains a significant amount of amorphous cellulose, which will likely undergo rapid thermal decomposition and produce a high yield of volatiles and bio-oil during the early stages of pyrolysis. The calculated HBI and TOI values are 0.750 and 1.401, respectively. The estimated HBI of 0.750 suggests a less rigid structure, which can translate to faster thermal degradation and a lower activation energy for the initiation of pyrolysis. Furthermore, the TOI value of 1.401 indicates a moderately organized lignocellulosic matrix. This combination of lower structural rigidity and moderate organization is conducive to high bio-oil yields (reported up to 45–50%) and high-quality char [78,79].

### 3.4. Analysis of TGA/DTG and Deconvoluted DTG Data

**Figure 4:**
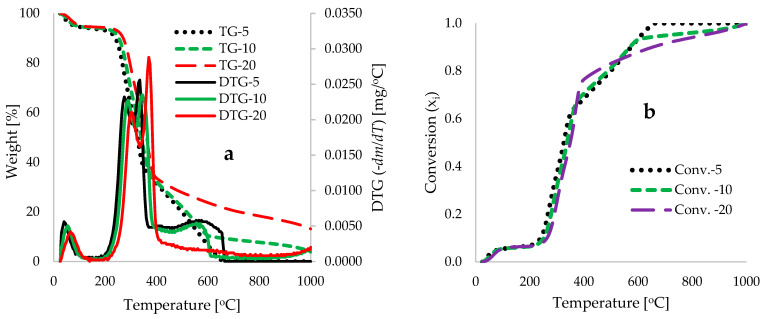

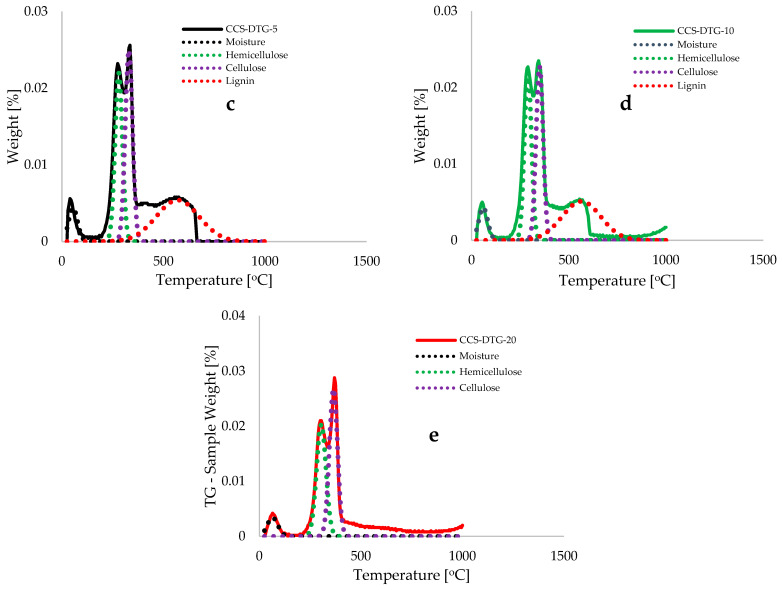
Graphs illustrating plots of TG and DTG profiles as a function of degradation temperature: (**a**) weight loss (wt%) and DTG (−dW/dT, [mg·°C^−1^]) at multiple heating rates; (**b**) extent of conversion (x_i_); and deconvoluted DTG curves at (**c**) 5 °C·min^−1^, (**d**) 10 °C·min^−1^, and (**e**) 20 °C·min^−1^.

Figure 4a presents the TG traces at varying heating rates of 5, 10, and 20 °C·min^−1^, with degradation temperatures ranging between 25 and 1000 °C. It is evident that the TG traces shift to higher temperature maxima with increasing heating rate, which can be attributed to the rate of heat transfer into the sample becoming limited by the rate of the chemical decomposition process itself [80]. A similar shift in temperature is seen with increasing heating rates in Figure 4a, which shows the DTG traces for the three heating rates. The TG/DTG experiment detailed in Figure 4a concluded at 1000 °C due to equipment temperature limitations. At elevated temperatures, typically above 600 °C, material degradation was still actively occurring for traces run at lower heating rates (5 and 10 °C·min^−1^). Conversely, traces at a higher heating rate of 20 °C·min^−1^ exhibited a nearly plateauing effect at these elevated temperatures. This observation suggests that decomposition reactions in this range were either approaching completion or that the rate-limiting step transitioned from a kinetically controlled process to a mass transfer-controlled process [81]. The plateau stage is considered the region of thermal stability, wherein no mass change occurs over time; consequently, it may not yield useful information for kinetic analysis [82,83].

Figure 4b presents the extent of conversion (x_i_) in relation to degradation temperature for the pyrolysis of CCS biomass, demonstrating its characteristic thermal decomposition across various material compositions and the slow mass conversion observed at elevated temperatures for the analyses conducted at high heating rate of 20 °C·min^−1^. Notably, the slow heating rate pyrolysis of this material at elevated temperatures resulted in further decomposition of the sample (significant mass loss), which can be ascribed to the longer residence time associated with slow pyrolysis at 5 and 10 °C·min^−1^, facilitating secondary reactions that further depolymerize the intermediate complex structures into smaller molecules [84]. This also suggests that the rate of conversion over time during the thermochemical conversion of the CCS biomass material is influenced by the heating rate, with the depolymerization of the hemicellulose and cellulose components occurring rapidly through the cleavage of glycosidic bonds, leading to the formation of levoglucosan (1,6-anhydro-β-D-glucopyranose) [85].

Figure 4c–e present the experimental and deconvoluted DTG profiles at heating rates of 5, 10, and 20 °C·min^−1^. These plots facilitate the characterization of peak widths, heights, and centers corresponding to the degradation of moisture, hemicellulose, and cellulose, alongside the extended decomposition of lignin at higher temperatures. The associated lignocellulosic compositions, temperature ranges, and bioproduct yields derived from the deconvoluted data are summarized in Table 3. The deconvoluted profiles indicate overlapping thermal degradation regions for moisture (25–160 °C), hemicellulose (135–395 °C), cellulose (270–430 °C), and lignin (275–675 °C). The broad degradation range observed for lignin, relative to the hemicellulose and cellulose fractions, is attributed to its complex, amorphous, three-dimensional aromatic framework, which comprises various chemical bonds with disparate thermal stabilities [86]. This aligns with findings by Yang et al. [66], who reported that hemicellulose and cellulose degrade within 220–315 °C and 315–400 °C, respectively, while the passive pyrolysis of the stable lignin structure spans 160–900 °C, Conversely, Rizal et al. [87] observed that *Cocos nucifera* shell decomposition began at 180 °C (hemicellulose) and 350 °C (cellulose), with lignin degrading between 300 and 500 °C.

Table 3 shows moisture contents derived from DTG deconvolution (5.2–5.8 wt%) were consistent with the proximate analysis results in Table 1 (6.3 wt%). Initial mass loss (up to 8 wt% conversion) is attributed to dehydration and the volatilization of low-molecular-weight phytochemicals, such as tannins and saponins [88,89], preceding rapid depolymerization. While Table 4 details compositions across all rates, the literature suggests that lower heating rates (5–10 °C·min^−1^) minimize thermal lag and improve peak resolution [90,91,92]. Notably, the lignin DTG peak is distinct at these lower rates but obscured at 20 °C·min^−1^ (Figure 4a). Consequently, the active pyrolysis stage of the CCS biomass was characterized by the degradation of hemicellulose (23.9–24.5 wt%), cellulose (25.4–27.1 wt%), and residual lignin (26.8–28.5 wt%).

Table 4 shows the calculated yields of bioproducts derived from deconvoluted TG data using the empirical relations in Equations (S3)–(S7). Under optimized slow-to-intermediate pyrolysis conditions (heating rates of 5–10 °C·min^−1^), the calculated yields range from 6.5–10.4 wt% biochar, 24.7–31.0 wt% bio-oil/light component (LC), 56.4–59.2 wt% bio-oil/gas-formation (GF), to 5.8–6.1 wt% syngas. The product distribution—characterized by moderate bio-oil, high liquid–gas mixture, and moderate-to-low char—is dictated by the chemical structure of the lignocellulosic biomass and specific reactor parameters. The high proportion of bio-oil/GF indicates that the low heating rate effectively facilitates the staged decomposition of hemicellulose and cellulose, while intermediate temperatures promote the maximum release of these volatile liquids/vapors, mitigating extensive secondary cracking into non-condensable gases. Furthermore, the calculated moderate bio-oil/LC yield indicates moderate secondary cracking reactions, forming light-weight molecules rather than heavy tars [93]. Consequently, the data indicates that this slow-intermediate pyrolysis process, utilizing controlled heating, optimizes liquid–gas production over solid char, consistent with the prior literature on coconut shell pyrolysis efficiency.

### 3.5. Model-Free and Model-Fitting Thermokinetic Data

**Figure 5:**
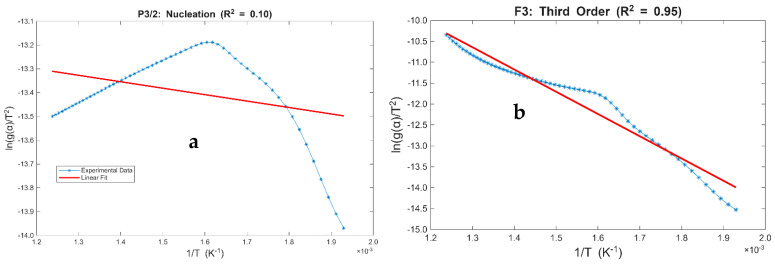

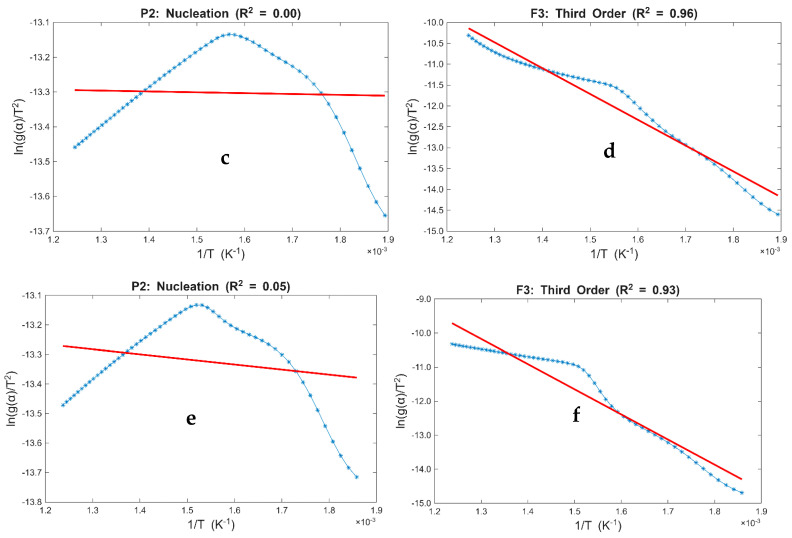
Graphs illustrating plots of Coats–Redfern $ln\left(\frac{g\left(x_{i}\right)}{T^{2}}\right)$ against the inverse of conversion temperature for the (**a**) nucleation [P3/2] at a heating rate of 5 °C·min^−1^; (**b**) Third Order [F3] at a heating rate of 5 °C·min^−1^; (**c**) nucleation [P2] at a heating rate of 10 °C·min^−1^; (**d**) Third Order [F3] at a heating rate of 10 °C·min^−1^; (**e**) nucleation [P2] at a heating rate of 20 °C·min^−1^, and (**f**) Third Order [F3] at a heating rate of 20 °C·min^−1^.

Figure 5 illustrates the Coats–Redfern (CR) model-fitting plots, depicting $ln\left(\frac{{g(x}_{i})}{T^{2}}\right)$ as a function of the inverse of conversion temperature for conversions between 10 and 85%. The upper limit of 85% was established due to the plateauing of the TG trace observed at 20 °C·min^−1^, which reached approximately 87% conversion at the 1000 °C maximum temperature threshold. Conversely, the 10% lower limit was selected to exclude initial mass loss associated with volatile impurities, ensuring the kinetic analysis reflects the intrinsic thermal degradation of the sample [94]. The kinetic parameters derived from the CR method at heating rates of 5, 10, and 20 °C·min^−1^ are summarized in Table 4. Evaluation of the reaction models indicates that the P3/2 (nucleation) model yields the poorest fit for the 5 °C·min^−1^ data, while the P2 (nucleation) model shows significant deviations at 10 and 20 °C·min^−1^. Conversely, the F3 reaction model (third order) consistently yielded the best fit formal kinetic model within the specified conversion range for all heating rates, achieving $R^{2\;}$ values of 0.95, 0.96, and 0.93, respectively.

While some biomass types exhibit nucleation-driven char formation, the complex structural matrix of coconut shell—characterized by robust lignin–lignin and lignin–cellulose linkages—is more accurately described by continuous degradation mechanisms. The F3 kinetic model suggests that pyrolysis is not driven by the physical growth of nuclei, but rather by the simultaneous, progressive breakdown of its three primary constituents. Lignin contributes to a protracted degradation profile that extends into higher temperature ranges, necessitating higher-order kinetic descriptions [95,96]. This consistency across various heating rates implies that while thermal lag shifts the reaction peaks, the underlying chemical decomposition mechanism remains unchanged. These findings align with the existing literature on high-lignin woody biomass, where nucleation models typically fail to represent the “distributed activation energy” inherent in complex lignocellulosic matrices [97,98].

**Figure 6:**
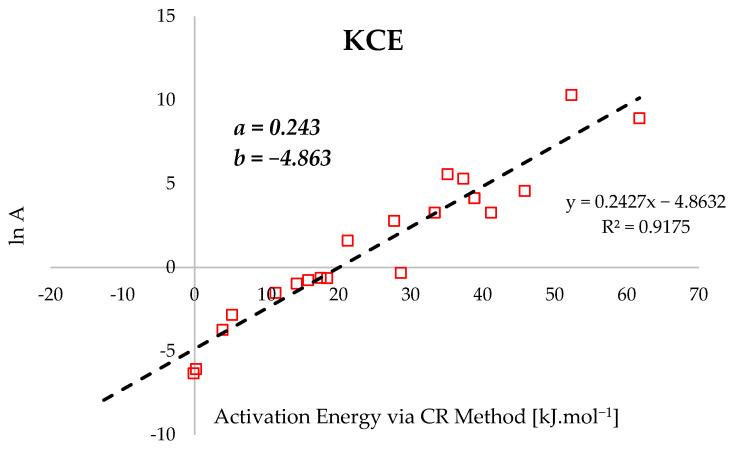
Kinetic compensation effect illustrating the plot of *lnA* against *E_CR_*.

Figure 6 illustrates the kinetic compensation effect (KCE) via the linear relationship between the natural logarithm of the pre-exponential factor (*lnA*) and the activation energy derived from the CR method. The regression analysis yields a slope (a) of 0.243 (a) and an intercept (b) of −4.863. This strong linear correlation suggests that despite the heterogeneous composition of the coconut shell—comprising hemicellulose, cellulose, and lignin—the pyrolytic conversion follows a consistent reaction mechanism [99,100].

The KCE parameters indicate a stable kinetic regime where the isokinetic temperature is governed by the slope, and the intercept represents the baseline frequency factor. This confirms that the various decomposition stages of the biomass components are kinetically interrelated [101,102]. The positive slope signifies that increases in the energy barrier are compensated by a higher frequency of molecular collisions and active site availability. Regarding valorization, the high volatile content (71.5%) and relatively low ash (9.2%) facilitate sustained reactivity [103]. Consequently, these constants suggest that the pyrolysis process remains mechanically uniform across varying operational conditions, providing a predictable framework for bioreactor design and industrial scaling.

**Figure 7:**
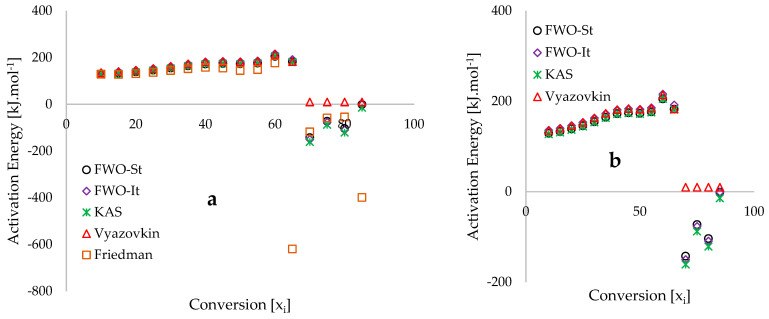
Plots of activation energy against the extent of conversion (x_i_ [%]) derived from the multiple isoconversional kinetic analyses for the selected (**a**) 5 isoconversional models and (**b**) excluding Friedman differential isoconversional method.

Figure 7 illustrates the activation energy profiles as a function of conversion ($x_{i}$), derived using several isoconversional methods: FWO-St, FWO-It, KAS, Vyazovkin, and Friedman. Within the $0.10\;\leq x_{i}\leq 0.65$ range, a consistent increase in activation energy is observed. However, beyond $x_{i}=0.65$, the models exhibit a sharp decline, yielding physically inconsistent negative activation energy values. This phenomenon is a known mathematical artifact arising from the overlapping conversion traces observed in Figure 4b at higher temperatures. This region corresponds to lignin degradation and secondary char reactions, which constitute approximately 30 wt% of the coconut shell (Table 3). At a heating rate of 20 °C·min^−1^, the reaction exhibits a lower relative rate or shifts toward lower temperatures due to complex thermal transitions. Consequently, the slopes of the linear isoconversional plots (FWO, KAS, and Friedman) become positive, while the Vyazovkin estimates drop significantly to $10\;kJ.{mol}^{-1}$. Such deviations highlight the limitations of applying Arrhenius kinetics to complex, non-Arrhenius systems where multiple simultaneous reactions occur [104,105].

To ensure kinetic reliability, the analysis focuses on the $0.10\;\leq x_{i}\leq 0.60$ range, representing the primary hemicellulose and cellulose decomposition stages (135–430 °C). In this interval, the four integral models (FWO-St, FWO-It, KAS, and Vyazovkin) show high convergence, with activation energy values ranging from 127.1 to 215.8 $kJ.{mol}^{-1}$. Conversely, the Friedman differential method exhibits significant divergence. This inconsistency is attributed to the model’s high sensitivity to experimental noise and its reliance on the derivative of conversion ($dx_{i}/dt$), which tends to amplify inaccuracies in heterogeneous biomass materials compared to the more stable mass loss data used by integral methods [106,107,108].

Table 5 summarizes the activation energy values derived from selected model-fitting and model-free isoconversional methods over a conversion range of 10–60 wt%. Utilizing recommended integral approaches—specifically FWO-St, FWO-It, KAS, and the Vyazovkin method—the mean activation energy data for this hemicellulose/cellulose fraction was determined to be 165 ± 5.2 kJ·mol^−1^, with a corresponding pre-exponential factor of 2.9 × 10^15^ min^−1^. These kinetic parameters offer critical insights into the pyrolytic mechanisms of coconut shells. The average activation energy of 165 kJ·mol^−1^ represents the mean energy barrier required for the thermal cleavage of chemical bonds within the lignocellulosic matrix. This value aligns with the moderate reactivity typically observed in high-performance lignocellulosic biomass. Although the results are consistent with the previously reported literature (Table 6), variations in activation energy are often associated with experimental differences in heating rates, sample mass, and the specific kinetic models employed. Consequently, kinetic data are heavily influenced by the experimental setup and the subsequent mathematical treatment [109,110]. Furthermore, the high magnitude of the pre-exponential factor suggests a significant frequency of effective molecular collisions, facilitating rapid bond dissociation and subsequent volatile matter evolution [111].

Table 6 presents the activation enthalpies calculated using both model-fitting and model-free kinetic methods. All derived values are positive, with the four integral isoconversional models yielding a mean value of 159 ± 5.2 kJ·mol^−1^. These enthalpies closely align with the estimated activation energies, maintaining a consistent difference of approximately 5.2 kJ·mol^−1^**.** This disparity corresponds to the magnitude of $R.T_{M}$, where R is the universal gas constant and $T_{M}$ is the peak temperature. The positive activation enthalpies suggest that the formation of activation complexes is an endothermic process, requiring a net input of energy to break the chemical bonds within the milled CCS biomass materials [112]. The small difference of 5.2 kJ·mol^−1^ between the overall activation energy and the activation enthalpy suggest that the process is thermodynamically favorable and can proceed once the energy barrier is met. This also correlates with an efficient devolatilization process of CCS biomass, meaning volatile components like organic acids, hydrocarbons, and alcohol are readily released during heating [3]. Furthermore, the calculated Gibbs free energy of activation yielded consistently positive values across all four integral isoconversional methods, with a mean value of 155 ± 1.4 kJ·mol^−1^. Based on literature-reported Gibbs free energy of activation values for hard woody biomass—including *Sterculia guttata* shell (152.1–158.8 kJ·mol^−1^) [113], *Melocanna baccifera* (165.3–193.7 kJ·mol^−1^) [114], Brazil nut residues (135.6–153.9 kJ·mol^−1^) [115], Poplar (157–187 kJ·mol^−1^) [116], and Oak (160–230 kJ·mol^−1^) [117]—the positive magnitudes of the Gibbs free energy of activation obtained for coconut shell pyrolysis in this study indicate that the formation of the activated complex is non-spontaneous, requires moderate energy, and is thermodynamically feasible. The comparative proximity of the estimated ${∆G}^{‡}$ to these lignocellulosic feedstocks highlights a similar, efficient reaction pathway for thermal degradation. The very low negative estimated values of entropy of activation in Table 6 signify that the activated complexes are highly ordered and structured compared to the reactants, classifying these reactions as slow [118]. Additionally, the estimated equilibrium constants for the formation of activation complexes in Table 7 are notably low and approach zero, suggesting that the thermochemical conversion of the CCS material strongly favors the reactants [42], indicating that the reactants are considerably more concentrated than the product at equilibrium.

### 3.6. Machine Learning kNN Modeling Data

Table 8 summarizes the optimized hyperparameters determined through a k-nearest neighbors (kNN) grid search, specifically identifying the optimal number of neighbors (k), distance metric, and weighting scheme for TG kNN modeling. The results indicate that a k-value of 85, Euclidean distance metric, and squared inverse weight scheme yielded the lowest validation Root Mean Squared Error (RMSE) of 0.646. Figure 4a shows that the thermal degradation of coconut shell involves complex, overlapping decomposition stages of hemicellulose, cellulose, and lignin. Consequently, the high optimal k-value (k=85) demonstrates that a larger neighborhood of data points improves model performance by capturing the consistent, underlying relationship between temperature and weight loss across the dataset, effectively filtering out minor experimental “noise” or fluctuations in the TG curves. The application of a squared inverse weight scheme, where closer neighbors have a greater influence, ensures the model remains sensitive to local thermal behavior variations while maintaining stability through a wide neighborhood perspective [121]. While several hyperparameter combinations yielded acceptable RMSE values (typically ≤1), the combination of k=1, Euclidean distance, and equal weighting—despite showing an RMSE <1—is not optimal for this application. A k=1 model acts as a direct look-up, leading to high-variance overfitting, whereby the model mimics experimental noise rather than generalizable thermal degradation trends. Therefore, the k=85 model offers superior generalization, crucial for predicting the pyrolysis behavior of coconut shell under varying operational conditions.

For DTG modeling, the combination of k=100, Euclidean distance, and a squared inverse weight scheme yielded the lowest validation RMSE (0.007) among the five randomly selected kNN subsets. The high k-value indicates that the DTG curve, representing the rate of mass loss, possesses a relatively smooth, global structure. In this context, a larger k acts as a low-pass filter, smoothing experimental noise or minor fluctuations in the degradation rates of hemicellulose, cellulose, and lignin. Furthermore, Euclidean distance is well-suited for continuous physical data where “straight-line” similarity between temperature-dependent mass loss rates is meaningful. The squared inverse scheme allows for 100 neighbors to be considered while ensuring closer neighbors in the “pyrolysis state” space exert greater influence, preserving local features—such as distinct DTG peaks—while benefiting from the stability of a large neighbor set.

Conversely, for conversion traces, the lowest RMSE (0.0104) was achieved with k=50, City Block distance and squared inverse weighting. This indicates that conversion traces require a more localized approach than DTG. Because conversion curves are cumulative, monotonic, and transition through specific kinetic regimes, they are highly sensitive to temperature-dependent Arrhenius-type behavior and phase-specific mechanisms [122,123,124]. Furthermore, City Block distance (also known as Manhattan or L_1_ distance) is more robust to outliers and “stepped” data patterns [125]. As shown in Figure 4b, coconut shell pyrolysis involves distinct “knees” between macromolecular components (e.g., cellulose to lignin), which City Block distance captures efficiently. Consequently, k=50 provides the sensitivity necessary to capture these local kinetic transitions, whereas the DTG modeling requires a broader, smoother approach.

**Figure 8:**
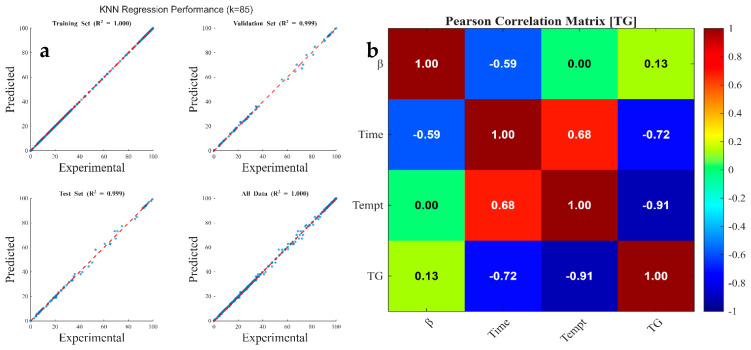
Graphical representations of (**a**) kNN-predicted TG data against experimental measurement for all data subset and (**b**) Pearson Correlation Matrix showing feature sensitivity to TG data.

**Figure 9:**
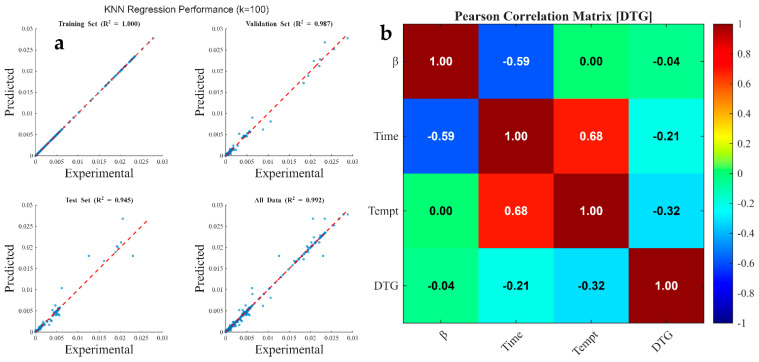
Graphical representations of (**a**) kNN-predicted DTG data against experimental measurement for all data subset and (**b**) Pearson Correlation Matrix showing feature sensitivity to DTG data.

**Figure 10:**
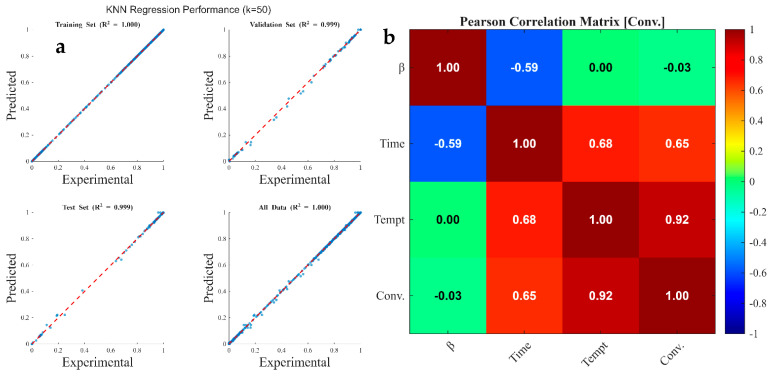
Graphical representations of (**a**) kNN-predicted conversion data against experimental measurement for all data subset and (**b**) Pearson Correlation Matrix showing feature sensitivity to Conversion data.

Figure 8, Figure 9 and Figure 10 illustrate the regression plots comparing kNN predictions against experimentally measured values for TG, DTG, and conversion traces, respectively. Additionally, the Pearson Correlation Matrix (Figure 8) delineates feature sensitivity regarding the predicted output. The kNN model achieved highly accurate predictions, with $R^{2}$ values approaching 1.000 for both TG and conversion datasets across all training, validation, and test subsets. For the DTG prediction, $R^{2}$ values were 1.000, 0.987, 0.945, and 0.992 for the 70% train, 15% validation, 15% test, and 100% total data subsets, respectively. These findings indicate that while kNN models effectively capture the integral, monotonic behavior of TG and conversion curves (high $R^{2}\approx 1.000$), they exhibit slightly lower performance when predicting the instantaneous rate of mass loss (DTG). The marginal decrease in $R^{2}$ during validation and testing for the DTG profiles (down to 0.945) highlights the algorithm’s sensitivity to the derivative nature of the data. Since the DTG curves (Figure 4a) contain sharp peaks and overlapping signals corresponding to complex biomass decomposition (hemicellulose, cellulose, and lignin), minor experimental noise in the TG data is amplified within the DTG domain.

In summary, the high accuracy in TG/conversion prediction stems from the smooth, consistent nature of high-density data, while the slight reduction in DTG test accuracy reflects the greater sensitivity of kNN to differential, noisy, and non-linear kinetic rates. This reliability is corroborated by the statistical error indices in Table 9, where the low MAE, MBE, MSE, and RMSE values indicate minimal dispersion and high model precision relative to experimental measurements.

Figure 8b, Figure 9b and Figure 10b illustrate the Pearson correlation matrices, quantifying the linear relationship between independent process parameters (heating rate, time, and temperature) and dependent thermogravimetric outputs (TG, DTG, and conversion). The Pearson correlation coefficient (r) serves as a metric for both the strength and direction of these associations, where values approaching ±1 indicate strong linearity [126]. The statistical analysis reveals that temperature and residence time are the primary determinants of mass degradation. For the TG output (Figure 8b), temperature exhibits a dominant strong negative correlation (r=−0.91), complemented by a significant negative correlation with time (r=−0.72). This inverse relationship confirms that as thermal energy and duration increase, the residual mass of the coconut shell decreases due to the volatilization of hemicellulose, cellulose, and lignin. Conversely, the conversion data (Figure 10b) shows strong positive correlations with temperature (r=0.92) and time (r=0.65), validating that these factors are the fundamental drivers for the transformation of solid biomass into volatile products [127].

In contrast to the cumulative metrics of TG and conversion, the DTG coefficients (Figure 9b) remain notably low, with values of −0.04 (heating rate), −0.21 (time), and −0.32 (temperature). These weak correlations indicate that the rate of mass loss (dm/dt) does not follow a simple linear progression with respect to the input variables. Instead, DTG peaks are governed by complex, stage-specific kinetic mechanisms—such as the rapid decomposition of specific polymers within narrow temperature windows—rather than a continuous linear acceleration. Across all outputs, the heating rate demonstrates negligible linear influence, with coefficients ranging from −0.04 to 0.13. This suggests that within the investigated experimental range, the magnitude of the heating rate is secondary to the absolute temperature achieved. While heating rate may influence the kinetics and heat transfer gradients, its statistical contribution to the overall variance in mass loss and conversion is minimal compared to the dominant effects of temperature and time. Overall, the Pearson correlation coefficients indicate that temperature and time are the dominant factors in coconut shell pyrolysis, directly impacting mass loss (TG) and conversion, while the heating rate has a negligible linear effect.

Though the present study successfully utilized the kNN model for predicting and optimizing CCS biomass thermal decomposition, few machine learning applications have tackled the complexity of biomass degradation processes like pyrolysis or torrefaction in the general literature. A notable exception is the work by Yin et al. [128], which deployed a BP-NN and a sophisticated stacked ensemble model (combining eXtreme Gradient Boosting, Light Gradient Boosting Machine, and a Multi-Layer Perceptron) for co-pyrolysis. That study reported excellent agreement with experimental data, with both models yielding R-squared values greater than 0.99. Pambudi et al. [9] evaluated multiple models (ANN, RF, DT, and kNN) for predicting the thermogravimetric data of oxidatively torrefied spent coffee grounds (SCGs). Their findings indicated that the kNN model outperformed the others, yielding R-squared values exceeding 0.99 for both mass loss (TG) and derivative mass loss (DTG) predictions. Complementing this, Otaru et al. [129] employed a k-nearest neighbors (kNN) model to successfully predict TGA traces for date palm waste pyrolysis, achieving a high R-squared value of 0.975 and identifying degradation temperature as the primary operational driver.

It is important to emphasize that the kNN modeling approach demonstrated high fidelity in capturing data subsets. Furthermore, the average activation energy (165 ± 5.2 kJ·mol^−1^), which is consistent with the standard literature for lignocellulosic biomass. However, the inherent complexity of coconut shell pyrolysis—specifically the extensive thermal stable degradation of high lignin content—resulted in the kinetic and thermodynamic parameters of the activated complex being statistically representative only within the 10 to 60 wt% conversion range. This interval primarily characterizes the active pyrolysis stage, dominated by hemicellulose and cellulose decomposition. To resolve the overlapping lignin degradation profiles, future research will utilize significantly lower heating rates ($\beta <10\;{}^{\circ}C.{min}^{-1}$) to improve peak resolution, integrated with hybrid machine learning architectures for the multi-objective optimization of the pyrolysis thermokinetics.

## 4. Conclusions

This study provides a definitive framework for the valorization of *Cocos nucifera* (coconut) shells, bridging experimental material science with advanced computational modeling to accelerate the global transition toward sustainable bioenergy. The comprehensive characterization confirms that coconut shells are a premier feedstock, featuring high volatile matter (72.3%), significant carbon content (45.7%), and a complete absence of sulfur, ensuring clean energy production with minimal pollutant emissions. FTIR and SEM/XEDS analyses further reveal a robust, aromatic-rich structural matrix, specifically characterized by resilient lignin–cellulose linkages and the presence of endogenous potassium (4.27%) which acts as a natural catalyst during thermal degradation.

The thermogravimetric investigation (TG/DTG) successfully decoupled the complex pyrolysis process into its primary lignocellulosic constituents. Deconvolution of DTG traces identified specific degradation windows: hemicellulose (135–395 °C), cellulose (270–430 °C), and the broad, stable degradation of lignin (275–675 °C). Under optimized slow-to-intermediate pyrolysis conditions, the process yielded up to 31.0 wt.% bio-oil and 10.4 wt.% biochar, demonstrating high efficiency in volatile recovery. Reaction modeling identified the F3 (third order) mechanism as the best-fitting kinetic model within the studied conversion range. Achieving high coefficients of determination (up to 0.96), this model proves that pyrolysis proceeds via continuous, progressive degradation rather than discrete nucleation.

The thermokinetic analysis within the 10–60% conversion range established a mean activation energy of 165 ± 5.2 kJ·mol^−1^ and a pre-exponential factor of 2.9 × 10^15^ min^−1^. The positive activation enthalpy (159 kJ·mol^−1^) and Gibbs free energy of activation (155 kJ·mol^−1^) indicate an endothermic, non-spontaneous process. These values suggest moderate energy requirements consistent with literature data for various hardwood biomass materials.

The pioneering integration of kNN machine learning modeling achieved near-perfect predictive accuracy, with $R^{2}$ values approaching 1.000 for TG and conversion profiles. Optimized hyperparameters—specifically k=85 for TG, k=100 for DTG, and k=50 for conversion—demonstrated the model’s ability to filter experimental noise while capturing intricate kinetic transitions. While this study’s kinetic and thermodynamic assessments demonstrate the viability of coconut shell for bioenergy, the active pyrolysis phase—driven by cellulose and hemicellulose decomposition—requires more granular analysis. Therefore, future investigations will employ low-heating-rate TG analysis to mitigate confounding effects from lignin overlap, combined with hybrid artificial intelligence architectures to optimize the kinetic model.

## Figures and Tables

**Table 1:** Proximate and ultimate analysis of the milled CCS biomass.

Proximate Analysis				Ultimate Analysis				
Moisture [%]	Fixed Carbon [%]	Volatile Matter [%]	Ash [%]	Carbon [%]	Hydrogen [%]	Nitrogen [%]	Sulfur [%]	Oxygen [%]
6.3	17.8	72.3	3.6	45.7	4.9	2.8	0.0	46.6

**Table 2:** XEDS data of CCS biomass.

Element	C	O	Na	Mg	P	Cl	K	Ca	Total
Mass [%]	49.34 ± 0.19	39.05 ± 0.40	2.64 ± 0.07	0.40 ± 0.02	1.50 ± 0.04	0.27 ± 0.02	4.27 ± 0.08	2.53 ± 0.07	100
Atom [%]	59.46 ± 0.02	35.33 ± 0.37	1.66 ± 0.04	0.24 ± 0.01	0.70 ± 0.02	0.11 ± 0.01	1.58 ± 0.03	0.92 ± 0.02	100
Magnification is ×3000			Fitting ration = 0.2898			Acceleration voltage = 15 kV			

**Table 3:** Temperature and CCS biomass composition data.

β [^o^C·min^−1^]	Composition	Peak Centre (T_p_, ^o^C)	Initial Temperature (T_i_, ^o^C)	Final Temperature (T_f_, ^o^C)	ΔT = T_f_ − T_i_ (T_f_, ^o^C)	Weight [%]	Biochar Yield [%]	Bio-Oil Yield LC [%]	Bio-Oil Yield GF [%]	Syngas Yield [%]
5	Moisture	55	25	135	110	5.2	6.5	31.0	56.4	6.1
	Hemicellulose	280	135	335	200	23.9				
	Cellulose	330	280	415	135	27.1				
	Lignin	570	275	675	400	28.5				
10	Moisture	60	25	160	135	5.7	10.4	24.7	59.2	5.8
	Hemicellulose	290	160	350	190	24.5				
	Cellulose	350	270	420	150	25.4				
	Lignin	565	290	625	335	26.8				
20	Moisture	65	25	155	130	5.8	23.7	16.3	54.5	5.4
	Hemicellulose	305	165	395	230	32.9				
	Cellulose	365	290	430	140	37.5				
	Lignin	-	-	-	-	-				

**Table 4:** Tabulated kinetic data for the application of CR kinetic methods and the 36 selected solid-state reaction mechanisms.

	β = 5 °C·min^−1^			β = 10 °C·min^−1^			β = 20 °C·min^−1^		
Solid-State Reaction Models	R^2^	E_CR_ [kJ.mol^−1^]	A [min^−1^]	R^2^	E_CR_ [kJ.mol^−1^]	A [min^−1^]	R^2^	E_CR_ [kJ.mol^−1^]	A [min^−1^]
A3/2: Avrami (*n* = 1.5)	0.814	31.6	1.2 × 10^1^	0.833	37.0	7.1 × 10^1^	0.824	43.4	5.2 × 10^2^
A2: Avrami (*n* = 2)	0.278	3.4	8.1 × 10^−3^	0.430	5.1	3.3 × 10^−2^	0.508	7.2	1.3 × 10^−1^
A3: Avrami (*n* = 3)	0.109	−1.3	−1.4 × 10^−3^	0.002	−0.2	−4.7 × 10^−4^	0.051	1.1	7.2 × 10^−3^
A4: Avrami (*n* = 4)	0.601	−3.7	−2.5 × 10^−3^	0.435	−2.8	−4.5 × 10^−3^	0.196	−1.9	−7.0 × 10^−3^
Au: Prout-Tomkins	0.603	−11.9	−3.5 × 10^−4^	0.571	−12.2	−6.7 × 10^−4^	0.593	−13.8	−1.0 × 10^−3^
F1/3: One-Third Order	0.562	11.3	5.2 × 10^−2^	0.626	14.1	2.1 × 10^−1^	0.648	17.1	8.6 × 10^−1^
F3/4: Three-Quarter Order	0.684	15.0	6.2 × 10^−2^	0.729	18.2	2.7 × 10^−1^	0.735	22.0	1.2 × 10^0^
F3/2: One and Half Order	0.833	23.0	1.3 × 10^0^	0.855	27.4	6.4 × 10^0^	0.841	32.8	4.1 × 10^1^
F1: First Order	0.743	17.5	5.2 × 10^−1^	0.779	21.1	2.4 × 10^0^	0.777	25.3	1.2 × 10^1^
F2: Second Order	0.892	29.4	1.4 × 10^1^	0.904	34.6	8.3 × 10^1^	0.884	41.3	6.9 × 10^2^
F3: Third Order	0.951	44.2	6.4 × 10^2^	0.956	51.4	5.3 × 10^3^	0.929	61.4	8.3 × 10^4^
D1: 1D Diffusion	0.690	28.2	2.1 × 10^0^	0.721	33.2	1.2 × 10^1^	0.725	38.5	6.5 × 10^1^
D2: 2D Diffusion	0.743	33.0	3.9 × 10^0^	0.768	38.7	2.4 × 10^1^	0.767	44.8	1.6 × 10^2^
D3: 3D Diffusion (Jander)	0.797	39.1	4.3 × 10^0^	0.816	45.5	3.1 × 10^1^	0.810	52.8	2.5 × 10^2^
D4: Ginstling-Brounshtein	0.763	35.1	1.5 × 10^0^	0.786	40.9	9.5 × 10^0^	0.783	47.4	6.8 × 10^1^
D5: Zhuravlev, Lesokin, Tempelman	0.875	52.9	1.5 × 10^2^	0.885	61.1	1.5 × 10^3^	0.871	71.2	2.1 × 10^4^
D6: Anti-Jander	0.647	24.1	6.6 × 10^−2^	0.684	28.6	3.4 × 10^−1^	0.693	33.3	1.7 × 10^0^
R1: One-Dimension	0.445	8.7	3.3 × 10^−2^	0.527	11.2	1.3 × 10^−1^	0.563	13.8	4.9 × 10^−1^
R2: GCM (Contracting Cylinder)	0.614	12.7	6.2 × 10^−2^	0.670	15.7	2.5 × 10^−1^	0.685	19.0	1.1 × 10^0^
R3: GCM (Contracting Sphere)	0.662	14.2	6.5 × 10^−2^	0.710	17.4	2.8 × 10^−1^	0.719	20.9	1.2 × 10^0^
P1: Nucleation)	0.445	8.7	3.3 × 10^−2^	0.527	11.2	1.3 × 10^−1^	0.563	13.8	4.9 × 10^−1^
P3/2: Nucleation	0.101	2.3	3.1 × 10^−3^	0.221	3.9	1.4 × 10^−2^	0.310	5.6	5.4 × 10^−2^
P2: Nucleation	0.036	−1.0	−8.5 × 10^−4^	0.001	0.2	4.3 × 10^−4^	0.048	1.4	7.3 × 10^−3^
P3: Nucleation	0.575	−4.2	−2.2 × 10^−3^	0.438	−3.5	−4.1 × 10^−3^	0.270	−2.7	−7.3 × 10^−3^
P4: Nucleation	0.806	−5.9	−2.4 × 10^−3^	0.747	−5.3	−4.7 × 10^−3^	0.653	−4.8	−9.3 × 10^−3^
Carter	0.662	14.2	6.5 × 10^−2^	0.710	17.4	2.8 × 10^−1^	0.719	20.9	1.2 × 10^0^

**Table 5:** Estimated activation energy (*E*) values for the pyrolysis of CCS biomass at 10–60 wt% conversion.

	FWO-St		FWO-It		KAS		Vyazovkin		Friedman	
Conversion x_i_ [-]	R^2^	E [kJ.mol^−1^]	R^2^	E [kJ.mol^−1^]	R^2^	E [kJ.mol^−1^]	R^2^	E [kJ.mol^−1^]	R^2^	E [kJ.mol^−1^]
10	1.000	129.1	1.000	135.9	1.000	127.1	1.000	135.4	0.752	128.7
15	1.000	133.0	1.000	139.9	0.999	130.9	1.000	140.2	0.620	127.5
20	1.000	138.8	1.000	146.0	1.000	136.8	1.000	146.1	0.443	130.6
25	1.000	145.7	1.000	153.3	1.000	144.0	1.000	153.2	0.510	136.3
30	1.000	154.7	1.000	162.7	1.000	153.3	1.000	162.9	0.540	144.1
35	1.000	164.4	1.000	172.9	1.000	163.3	1.000	173.2	0.523	152.2
40	1.000	172.4	1.000	181.4	1.000	171.6	1.000	181.6	0.453	158.0
45	1.000	174.5	1.000	183.6	1.000	173.6	1.000	183.7	0.300	154.7
50	1.000	173.1	1.000	182.1	1.000	171.9	1.000	182.2	0.230	144.2
55	1.000	176.4	1.000	185.6	1.000	175.3	1.000	184.7	0.371	148.0
60	0.998	205.2	0.998	215.8	0.998	205.4	1.000	214.1	0.445	176.7

**Table 6:** Summary of thermokinetic parameters for the CCS biomass within the 10–60 wt% conversion range.

Model	R^2^	*E* [kJ.mol^−1^]	*A* [min^−1^]	∆$H^{‡}$ [kJ·mol^−1^]	∆$G^{‡}$ [kJ·mol^−1^]	∆$S^{‡}$ [kJ·mol^−1·^K^−1^]	*k* [-]
CR	0.9452	52.3	29,810.5	47.1	173.4	−0.200	4.9 × 10^−15^
FWO-St	0.9997	160.7	7.0 × 10^14^	155.4	156.1	−0.001	1.3 × 10^−13^
FWO-It	0.9997	169.0	5.3 × 10^15^	163.8	153.7	0.016	2.1 × 10^−13^
KAS	0.9997	159.4	5.1 × 10^14^	154.1	156.4	−0.004	1.2 × 10^−13^
Vyazovkin	0.9998	168.8	5.1 × 10^15^	163.6	153.8	0.015	2.1 × 10^−13^
Friedman	0.4716	145.5	1.8 × 10^13^	140.3	160.3	−0.032	6.0 × 10^−14^

**Average:** *E* = 165 ± 5.2 kJ·mol^−1^; *A* = 2.9 × 10^15^ min^−1^; ${∆H}^{‡}$ = 159 ± 5.2 kJ·mol^−1^; ${∆G}^{‡}\;$= 155 ± 1.4 kJ·mol^−1^; ${∆S}^{‡}\;$= 0.007 kJ·mol^−1^.K^−1^; *k* = 1.7 × 10^−13^ [-].

**Table 7:** Kinetic parameters for coconut shell pyrolysis.

Source	Conversion	Kinetic Method	Heating Rate [^o^C·min^−1^]	Temperature [^o^C]	E [kJ.mol^−1^]	A [min^−1^]
Current	0.1–0.6	FWO-St, FWO-It, KAS, & Vyazovkin	5, 10, and 20	25–1000	165	2.90 × 10^15^
Said et al. [119]	Main Pyrolysis	CR	10	227–727	122.8	1.31 × 10^11^
Agrizzi et al. [104]	0.1–0.9	FWO and KAS	5, 10, and 20	30–1000	182.8–192.4	5.2 × 10^14^
Mian et al. [46]	0.1–0.8	CR	3, 5, and 10	25–900	159.6–177.5	8.4 × 10^12^
Tsamba et al. [98]	0.2–0.8	Isoconversional	20	250–400	180–216	1.5 × 10^15^
C. Cheng et al. [120]	0.1–0.7	Isoconversional	10, 15, and 20	150–600	194.5–238.3	4.7 × 10^16^

**Table 8:** Optimized kNN hyperparameter tuning.

**TG**					
**Simulation**	***k*** **-value**	**Distance**	**Weight**	**Val RMSE**	**Remark**
1	100	Euclidean	Squared Inverse	1.0570	✓
2	100	City Block	Squared Inverse	0.8351	✓
3	**85**	**Euclidean**	**Squared Inverse**	**0.6458**	✓✓
4	1	Euclidean	Equal	0.9119	×
5	50	Euclidean	Squared Inverse	0.7580	✓
**DTG**					
**Simulation**	***k*** **-value**	**Distance**	**Weight**	**Val RMSE**	**Remark**
1	85	Euclidean	Squared Inverse	0.0011	✓
2	20	City Block	Inverse	0.0009	✓
3	15	Euclidean	Squared Inverse	0.0009	✓
4	100	Euclidean	Squared Inverse	0.0007	✓✓
5	5	Euclidean	Squared Inverse	0.0012	✓
**Conversion**					
**Simulation**	***k*** **-value**	**Distance**	**Weight**	**Val RMSE**	**Remark**
1	1	Euclidean	Equal	0.0128	×
2	100	City Block	Squared Inverse	0.0120	✓
3	3	Euclidean	Inverse	0.0089	✓
4	5	Euclidean	Inverse	0.0108	✓
5	**50**	**City Block**	**Squared Inverse**	**0.0104**	✓✓

× means Not Recommended; ✓ means Recommended; ✓✓ mean Highly Recommended.

**Table 9:** Estimated measures of dispersion for the TG, DTG, and conversion prediction for all data subsets.

	**TG**			
**Performance Metrics**	**Train**	**Validation**	**Test**	**Overall**
Std Dev	8.73 × 10^−16^	0.649	1.006	0.462
MAE	1.62 × 10^−16^	0.363	0.523	0.133
MBE	−1.62 × 10^−16^	0.021	−0.071	−0.007
MSE	7.87 × 10^−31^	0.417	1.006	0.213
RMSE	8.87 × 10^−16^	0.646	1.003	0.462
R^2^	1.000	0.999	0.999	1.000
	**DTG**			
**Performance Metrics**	**Train**	**Validation**	**Test**	**Overall**
Std Dev	1.71 × 10^−19^	7.22 × 10^−4^	1.47 × 10^−3^	6.35 × 10^−4^
MAE	9.21 × 10^−21^	3.07 × 10^−4^	5.65 × 10^−4^	1.30 × 10^−4^
MBE	−8.16 × 10^−21^	−6.82 × 10^−5^	−2.35 × 10^−4^	−4.54 × 10^−5^
MSE	2.94 × 10^−38^	5.20 × 10^−7^	2.18 × 10^−6^	4.05 × 10^−7^
RMSE	1.71 × 10^−19^	7.21 × 10^−4^	1.48 × 10^−3^	6.36 × 10^−4^
R^2^	1.000	0.987	0.945	0.992
	**Conversion**			
**Performance Metrics**	**Train**	**Validation**	**Test**	**Overall**
Std Dev	0.000	1.01 × 10^−2^	1.20 × 10^−2^	6.13 × 10^−3^
MAE	0.000	5.56 × 10^−3^	7.33 × 10^−3^	1.93 × 10^−3^
MBE	0.000	−2.84 × 10^−3^	−9.91 × 10^−4^	−5.74 × 10^−4^
MSE	0.000	1.09 × 10^−4^	1.44 × 10^−4^	3.79 × 10^−5^
RMSE	0.000	1.04 × 10^−2^	1.20 × 10^−2^	6.15 × 10^−3^
R^2^	1.000	0.999	0.999	1.000

## Data Availability

The original contributions presented in this study are included in the article/Supplementary Materials. Further inquiries can be directed to the corresponding author.

## Supplementary Materials

The following supporting information can be downloaded at https://www.mdpi.com/article/10.3390/polym18091070/s1.
