# Supplementary material for: Comparative Reaction Modelling and k-Nearest Neighbors Analysis of Cocos nucifera Shell Thermal Degradation
Source: Polymers (Basel). 2026 Apr 28;18(9):1070. doi: 10.3390/polym18091070 (PMC13165420; doi:10.3390/polym18091070)
Supplement: Supplementary file 1 [file polymers-18-01070-s001.zip › polymers-4197388-supplementary.pdf]

Simpson 1/3rd rule

$$A_{CURVE} = \frac{h}{3}\left[f(x_0) + 4\sum_{1,3,5,\ldots}^{n-1} f(x_i) + 2\sum_{2,4,6,\ldots}^{n-2} f(x_j) + f(x_n)\right] \quad \text{(S1)}$$

where the width of each subinterval is calculated as $h = \frac{b-a}{n}$ (S2)

$f(x_0), f(x_i), f(x_j)$, and $f(x_n)$ are functions at start, odd numbers, even numbers, and at the end, respectively, and n is the number of datapoints.

TGA Data Analysis Steps

Moisture Removal: Ambient to ~ 150 °C (S3)

Bio-oil Calculation: The major weight loss after moisture removal (> 150 °C) up to 600 °C is generally assumed to be bio-oil.

Volatiles (Bio-oil precursors/Light components) % = $\left(\frac{Weight\ at\ 150\ ^{o}C - Weight\ at\ 300\ ^{o}C}{Initial\ Weight}\right)$ x 100 (S4)

Active Pyrolysis (Bio-oil/Gas formation) % = $\left(\frac{Weight\ at\ 300\ ^{o}C - Weight\ at\ 600\ ^{o}C}{Initial\ Weight}\right)$ x 100 (S5)

Biochar Calculation: The final weight at the end of the pyrolysis run (before oxygen is added represents the biochar yield.

Bio-char % = $\left(\frac{Weight\ at\ 600\ ^{o}C}{Initial\ Weight}\right)$ x 100 (S6)

Syngas Calculation: The remaining weight loss at high temperature (> 600 °C), which is not captured as liquid, represents syngas.

Syngas % = $100\% - (\text{Bio} - \text{oil}\% + \text{Syngas}\ \%)$ (S7)

Coats-Redfern (CR) Model-Fitting Kinetic Method

$$\ln\left(\frac{g(x_i)}{T^2}\right)_{x_i,i} = \ln\left(\frac{A_{MF}\cdot R}{\beta . E_{CR}}\right) - \left(\frac{E_{MF}}{R}\right)\cdot\frac{1}{T} \quad \text{(S8)}$$

$$A_{MF} = \frac{\beta\cdot E_{CR}}{R}\cdot \exp\text{ (Intercept)} \quad \text{(S9)}$$

where $g(x_i) = \int_0^{x_i}\frac{dx_i}{f(x_i)}$, R is the gas constant, T is the temperature, x signifies the conversion, E_{MF} is the model-fitting activation energy, A_{MF} is the model-fitting pre-exponential factor.

Kinetic Compensation Effect

$lnA_{MF} = aE_{CR} + b$ (Model-fitting) (S10)

$\ln A = aE + b$ (Model-free) (S11)

Table S1. Selected solid-state reaction mechanisms.

Reaction Mechanisms	$g(\alpha_i)$	$f(\alpha_i)$
**Geometrical Contraction Models (GCM)**		
One-dimension [R1]	α_i	1
Contracting sphere [R2]	$1-(1-\alpha_i)^{1/2}$	$(2)\ (1-\alpha_i)^{1/2}$
Contracting cylinder [R3]	$1-(1-\alpha_i)^{1/3}$	$(3)\ (1-\alpha_i)^{2/3}$
**Reaction-Order Models (ROM)**		
First-order reaction model [F1] or Mampel model	$-\ln(1-\alpha_i)$	$(1-\alpha)$
Second-order reaction model [F2]	$(1-\alpha_i)^{-1}-1$	$(1-\alpha_i)^2$
Third-order reaction model [F3]	$[(1-\alpha_i)^{-2}-1]/2$	$(1-\alpha_i)^3$
One-third order [F1/3]	$1-(1-\alpha_i)^{2/3}$	$(3/2)\ (1-\alpha_i)^{1/3}$
Three-quarters order [F3/4]	$1-(1-\alpha_i)^{1/4}$	$(4)\ (1-\alpha_i)^{4/3}$
Three-halves order [F3/2]	$(1-\alpha_i)^{-1/2}-1$	$(2)\ (1-\alpha_i)^{3/2}$
**Power Law Models (PLM)**		
Power law [P1]	α_i	1
Power law [P3/2]	${\alpha_i}^{2/3}$	$(2/3)\ {\alpha_i}^{-1/2}$
Power law [P2]	${\alpha_i}^{1/2}$	$2{\alpha_i}^{1/2}$
Power law [P3]	${\alpha_i}^{1/3}$	$3{\alpha_i}^{2/3}$
Power law [P4]	${\alpha_i}^{1/4}$	$4{\alpha_i}^{3/4}$
**Diffusion Models (DFM)**		
1D Diffusion model [D1]	${\alpha_i}^2$	$1/(2\alpha_i)$
2D Diffusion model [D2]	$[(1-\alpha_i)\ln(1-\alpha_i)]+\alpha_i$	$[-\ln(1-\alpha_i)]^{-1}$
3D Diffusion model (Jander [D3])	$[1-(1-\alpha_i)^{1/3}]^2$	$(3/2)\ (1-\alpha_i)^{2/3}/[(1-(1-\alpha_i)^{1/3})]$
Ginstling-Brounshtein [D4]	$1-(2\alpha_i/3)-(1-\alpha_i)^{2/3}$	$(3/2)/[((1-\alpha_i)^{-1/3}-1)]$
Zhuravlev, Lesokin, Tempelman [D5]	$[(1-\alpha_i)^{-1/3}-1]^2$	$(3/2)\ (1-\alpha_i)^{4/3}/[((1-\alpha_i)^{-1/3}-1)]$
Anti-Jander [D6]	$[(1+\alpha_i)^{1/3}-1]^2$	$(3/2)\ (1+\alpha_i)^{2/3}/[((1+\alpha_i)^{1/3}-1)]$
**Sigmoidal Rate Equations (SRE)**		
Avarami-Erofeev [A3/2]	$[-\ln(1-\alpha_i)]^{3/2}$	$(3/2)\ (1-\alpha_i).[-\ln(1-\alpha_i)]^{1/3}$
Avarami-Erofeev [A2]	$[-\ln(1-\alpha_i)]^{1/2}$	$(2)\ (1-\alpha_i).[-\ln(1-\alpha_i)]^{1/2}$
Avarami-Erofeev (A3)	$[-\ln(1-\alpha_i)]^{1/3}$	$(3)\ (1-\alpha_i).[-\ln(1-\alpha_i)]^{2/3}$
Avarami-Erofeev [A4]	$[-\ln(1-\alpha_i)]^{1/4}$	$(4)\ (1-\alpha_i).[-\ln(1-\alpha_i)]^{3/4}$
Prout-Tomkins [Au]	$(\alpha_i)\ (1-\alpha_i)$	$\ln[\alpha_i/(1-\alpha_i)]$
**Carter Model (CM)**	$[1-(1-\alpha_i)^{1/3}]$	$(3/2)\ (1-\alpha_i)^{2/3}\ [(1-(1-\alpha_i)^{1/3})]^{-1}$

Model-Free Isoconversional Kinetic Methods

Standard Flynn-Wall-Ozawa (FWO-St) Method

$$\ln\ \beta = \ln\left(\frac{A\cdot E}{R\cdot(g(\alpha_i))}\right) - 5.331 - 1.052\left(\frac{E}{R}\right)\cdot\frac{1}{T} \qquad \text{(S12)}$$

Iterative Flynn-Wall-Ozawa (FWO-Ir) Method

$$\ln\ [\beta/M(x)] = \ln\left(\frac{0.0048\ A.E}{R.(g(\alpha_i))}\right) - 1.052\left(\frac{E}{R}\right).\frac{1}{T} \qquad \text{(S13)}$$

$$M(x) = \frac{\exp(-x).h(x)/x^2}{0.0048\exp(-1.052x)};\ x = \left(\frac{E}{R}\right).\frac{1}{T}\ \text{and}\ h(x) = \frac{x^4+18x^3+88x^2+96x}{x^4+20x^3+120x^2+240x+120} \qquad \text{(S14)}$$

Kissinger-Akahira-Sunose (KAS) Method

$$\ln\ [\beta/T^2] = \ln\left(\frac{A.R}{E\cdot(g(\alpha_i))}\right) - \left(\frac{E}{R}\right)\cdot\frac{1}{T} \qquad \text{(S15)}$$

where β is the heating rate, E is the activation energy, A is the frequency or pre-exponential factor, R is the gas constant, $g(x_i)$ is the integral form of the kinetic model, T is the temperature, x is the dimensionless variable representing the ration of the activation energy to the thermal energy, $h(x)$ is the Senum and Yang 4th - degree approximation.

Vyazovkin Method (VYM)

$$\frac{dx_i}{dt} = A \cdot \exp\left(-\frac{E_{\alpha_i}}{RT_\varepsilon}\right) \cdot f(\alpha_i) \quad \text{(S16)}$$

Rearranging Eqn S5.4 and integrating leads to the Eqn S5.5

$$\int_0^{\alpha_i} \frac{d\alpha_i'}{f(\alpha_i')} = \frac{A_i}{\beta_{Ri}} \int_{T_{i,0}}^{T_{i,\alpha_i}} \exp\left(-\frac{E_{\alpha_i}}{RT_\varepsilon}\right) dT = \frac{A_i}{\beta_{Rj}} \int_{T_{j,0}}^{T_{j,\alpha_i}} \exp\left(-\frac{E_{\alpha_i}}{RT_\varepsilon}\right) dT \quad \text{(S17)}$$

T_{i,α_i} and T_{j,α_i} are the conversion temperatures for the two experiments i and j, and β_{Ri} and β_{Rj} are the heating rates for the two experiments i and j.

Friedman (FR) Method

$$\ln\left(\frac{d\alpha_i}{dt}\right)_{\alpha_i,i} = \ln\left(A_{\alpha_i} \cdot f(\alpha_i)\right) - \left(\frac{E}{R}\right) \cdot \frac{1}{T_{\alpha_i,i}} \quad \text{(S18)}$$

<u>Thermodynamic Equations</u>

Entropy of activation	$\Delta S^{\ddagger} = R.\ln\left(\frac{A.h}{B_K.T_{CM}}\right)$	(S19)
Activation enthalpy	$\Delta H^{\ddagger} = E - R.T_{CM}$	(S20)
Gibbs free energy of the activation	$\Delta G^{\ddagger} = \Delta H^{\ddagger} - T_{CM}.\Delta S^{\ddagger}$	(S21)
Equilibrium constant	$k = \exp\left(-\Delta G^{\ddagger}/(E - \Delta H^{\ddagger})\right)$	(S22)

where T_{CM} is the temperature of maximum conversion, B_K signifies the Boltzmann constant (1.3806 x 10^{-23} J.K^{-1}), and h denotes the Planck's constant (6.626 x 10^{-34} J.s^{-1}).
